# Platelet-rich Plasma and Mesenchymal Stem Cells Local Infiltration Promote Functional Recovery and Histological Repair of Experimentally Transected Sciatic Nerves in Rats

**DOI:** 10.7759/cureus.8262

**Published:** 2020-05-24

**Authors:** Nikolaos Kokkalas, Panagiotis Kokotis, Kalliopi Diamantopoulou, Antonios Galanos, Pavlos Lelovas, Dionysios J Papachristou, Ismene A Dontas, Ioannis K Triantafyllopoulos

**Affiliations:** 1 Orthopaedics, Laboratory for Research of the Musculoskeletal System, KAT General Hospital, Medical School, National & Kapodistrian University of Athens, Athens, GRC; 2 Neurology, Laboratory of Clinical Neurophysiology, Aeginitio Hospital, Medical School, National & Kapodistrian University of Athens, Athens, GRC; 3 Pathology, KAT General Hospital, Athens, GRC; 4 Epidemiology and Public Health, Laboratory for Research of the Musculoskeletal System, KAT General Hospital, Medical School, National & Kapodistrian University of Athens, Athens, GRC; 5 Veterinary Medicine, Laboratory for Research of the Musculoskeletal System, KAT General Hospital, Medical School, National & Kapodistrian University of Athens, Athens, GRC; 6 Pathology, Unit of Bone and Soft Tissue Studies, Laboratory of Anatomy-Histology-Embryology, School of Medicine, University of Patras, Patras, GRC; 7 Pathology, School of Medicine, University of Pittsburgh, Pittsburgh, USA; 8 Orthopaedics, HYGEIA Private Hospital, Athens, GRC

**Keywords:** plateler rich plasma, mesenchymal stem cells, nerve repair, nerve regeneration

## Abstract

Introduction

Platelet-rich plasma (PRP) products and mesenchymal stem cells (MSCs) seem to have a significant potential as neurogenic therapeutic modulator systems. This study aimed to investigate such biological blood derivatives that could enhance nerve regeneration when applied locally in the primary repair of peripheral nerve transection of an experimental rat model.

Methods

A total of 42 two-month-old male Wistar rats were divided into three “treatment” groups (control, PRP, and MSCs). All the subjects were operated under anesthesia, and the surgical site was infiltrated with either normal saline, PRP derived from the animal’s peripheral blood, or MSCs derived from the animal’s femoral bone marrow. All three groups were also sub-divided into two sub-groups based on the post-operative administration of Non-steroidal anti-inflammatory drugs (NSAIDs) or not in order to evaluate the effect of NSAIDs on the final outcome. Three months post-surgery, electromyography evaluation of both hind limbs (right operated and left non-operated) was performed. The animals were euthanized, and nerve repair specimens were prepared for histology.

Results

PRP group had a significant effect (p<0.05) on the sciatic nerve repair when compared with the control group, whereas the MSC group had a positive effect but was not statistically significant (p=0.2). The number of counted neural axons at the area distal to the nerve repair site were significantly repetitive (p<0.05) in both the PRP and MSC groups when compared with the control group.

Conclusions

Both PRP and MSCs appear to play an essential role in the enhancement of nerve repair in terms of functionality and histology. MSCs group demonstrated a positive effect, whereas the PRP group showed statistically significant better results.

## Introduction

Side-to-side tension-free micro-surgical repair or transplantation of a nerve autograft to bridge a nerve gap remains the golden standard technique for the enhancement of the intrinsic regenerative potential of injured neuronal axons [1]. However, such treatments do not recreate the suitable cellular and molecular micro-environment for a satisfactory regeneration. Thus, recovery of such nerve injuries is incomplete [2].

In adjuvant biological treatment that would enhance nerve regeneration and improve nerve function, local application of platelet-rich plasma (PRP) derivatives and pure mesenchymal stem cells (MSCs) could be promising interventions in addition to the nerve repair. PRP products hold an important therapeutic potential as neuroprotective and neurogenic modulator systems [3]. In the literature, the results of PRP use are contradictory; there are studies supporting their boosting effects on nerve repair, whereas others put it in doubt [4].

MSCs, either derived from the bone marrow or adipose tissue, appear to enhance axon regeneration [5]. They produce this positive effect not only when delivered to the injured nerve or conduit bridging the nerve gap but also when administered intravenously. The MSCs’ migration potential made their detection possible at the site of sciatic nerve injury on day 7 post-intravenous injection to mice and enhanced the functional recovery of the sciatic nerve [6,7]. However, this observation was not in agreement with the findings of another study in a rat sciatic nerve injury model, where MSCs combined with a fibrin glue conduit promoted axon regeneration only when exposed to immunosuppressive treatment with cyclosporine A [6].

Therefore, the impact of biological agents on nerve regeneration continues to be a field of interest. The aim of this experimental animal study was to show the effect of PRP and bone-derived MSCs (b-MSCs) on the enhancement of nerve regeneration when locally applied in the primary repair of peripheral sciatic nerve transection using an experimental animal model. The originality of this study is the comparison of electromyography (EMG) results between the operated leg and the non-operated one, as well as the comparison of the histopathological results of the injured nerve distal and proximal with respect to the repair. In addition, the role of intramuscularly administered NSAIDs on the final outcome of nerve repair was also evaluated.

## Materials and methods

The protocol was approved by the Directorate of Veterinary Services of Prefecture of Athens, Attica, Greece, according to Greek legislation regarding ethical and experimental procedures (EL 25 BIO 018). Forty-two two-month-old male Wistar albino rats weighing between 200 and 240 g were included in the study. The rats were randomly divided into three “treatment” groups: (1) control group, where nerve repair was only performed, (2) the PRP group, where autologous PRP was isolated from the peripheral vein blood and locally infiltrated into the nerve repair, and (3) the MSCs group, where b-MSCs obtained from the ipsilateral femoral bone marrow were locally applied onto the nerve repair area. Each “treatment” group was further sub-divided into two “anti-inflammatory” sub-groups: (1) the with-NSAIDs group, where daily administration of NSAIDs was performed for 7 days post-operatively, and (b) the without-NSAIDs group, where NSAIDs were not administered (Table 1).

**Table 1:** “Treatment” groups and “anti-inflammatory” sub-groups. PRP, platelet-rich plasma; MSCs, mesenchymal stem cells; P, paracetamol; C, control; NSAIDs, non-steroidal anti-inflammatory drugs

“Treatment” groups	C (nerve repair only)	PRP (nerve repair + PRP infiltration)	MSCs (nerve repair + bone derived MSCs infiltration)	
			“Anti-inflammatory” sub-groups			
P only	7 (C - P)	7 (PRP - P)	7 (MSCs - P)	
NSAIDs and P	7 (C - NSAIDs + P)	7 (PRP - NSAIDs + P)	7 (MSC - NSAIDs + P)	

Surgical technique

**Figure 1:**
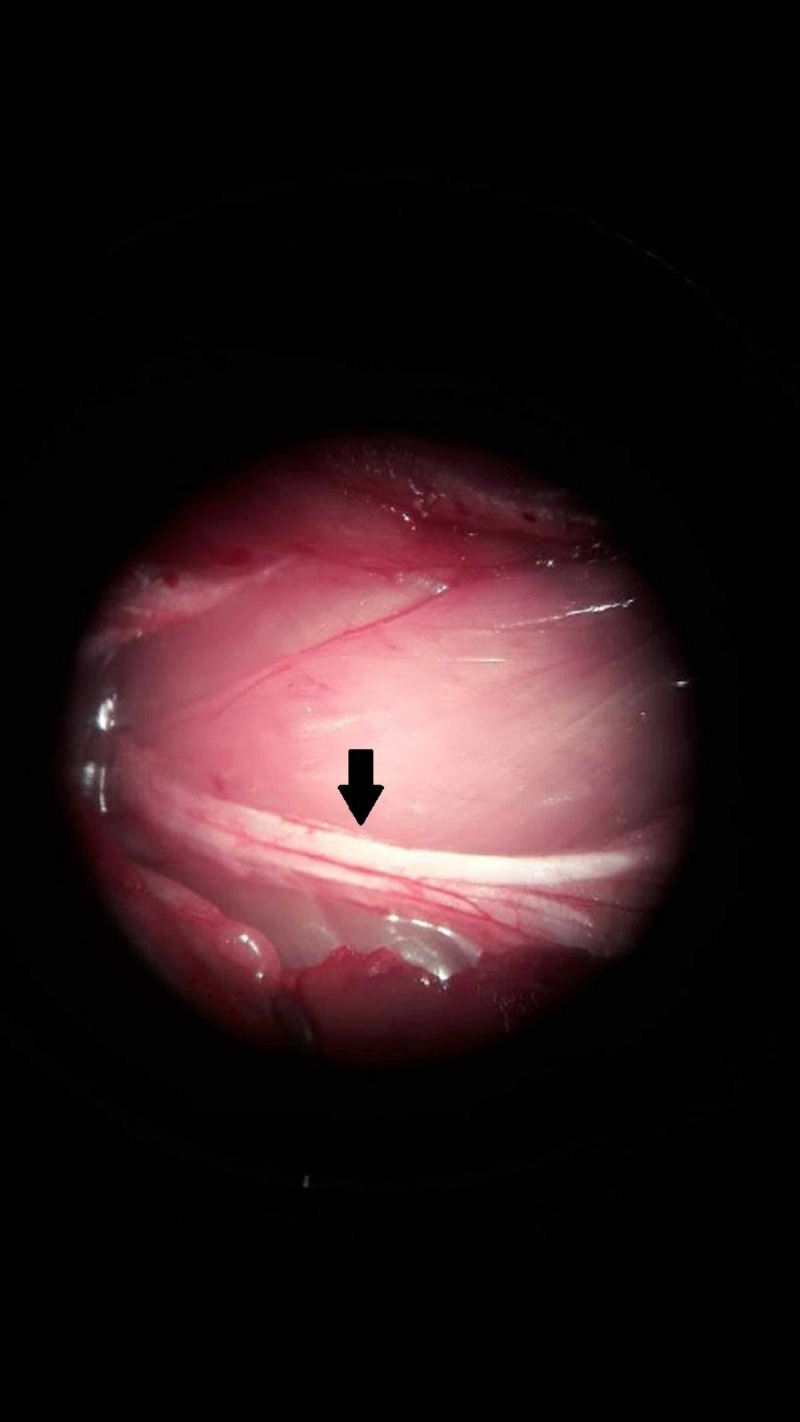
Sciatic nerve (black arrow) of the rat before transection (microscopy x16).

**Figure 2:**
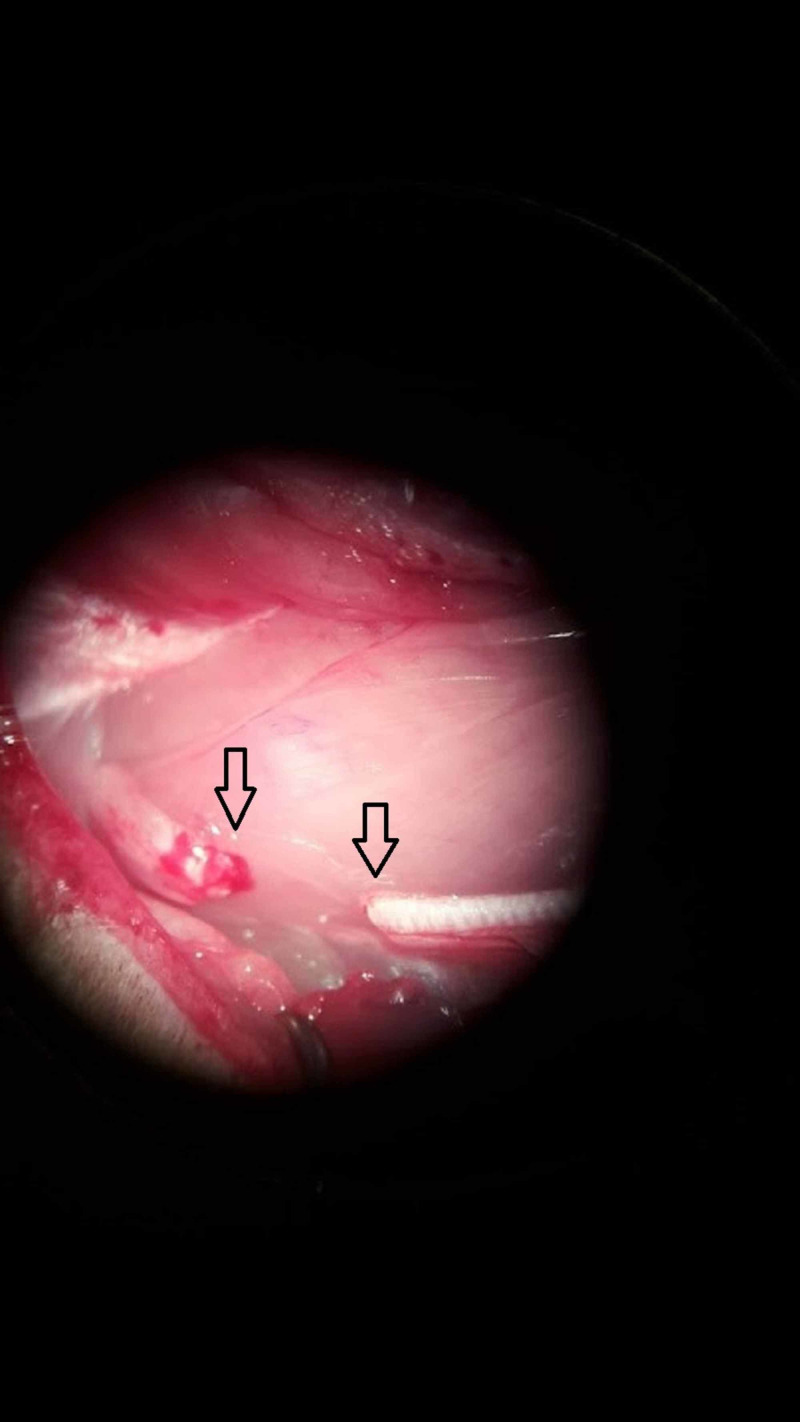
Sciatic nerve of the rat after transection (microscopy x16). The two arrows show the transected ends.

**Figure 3:**
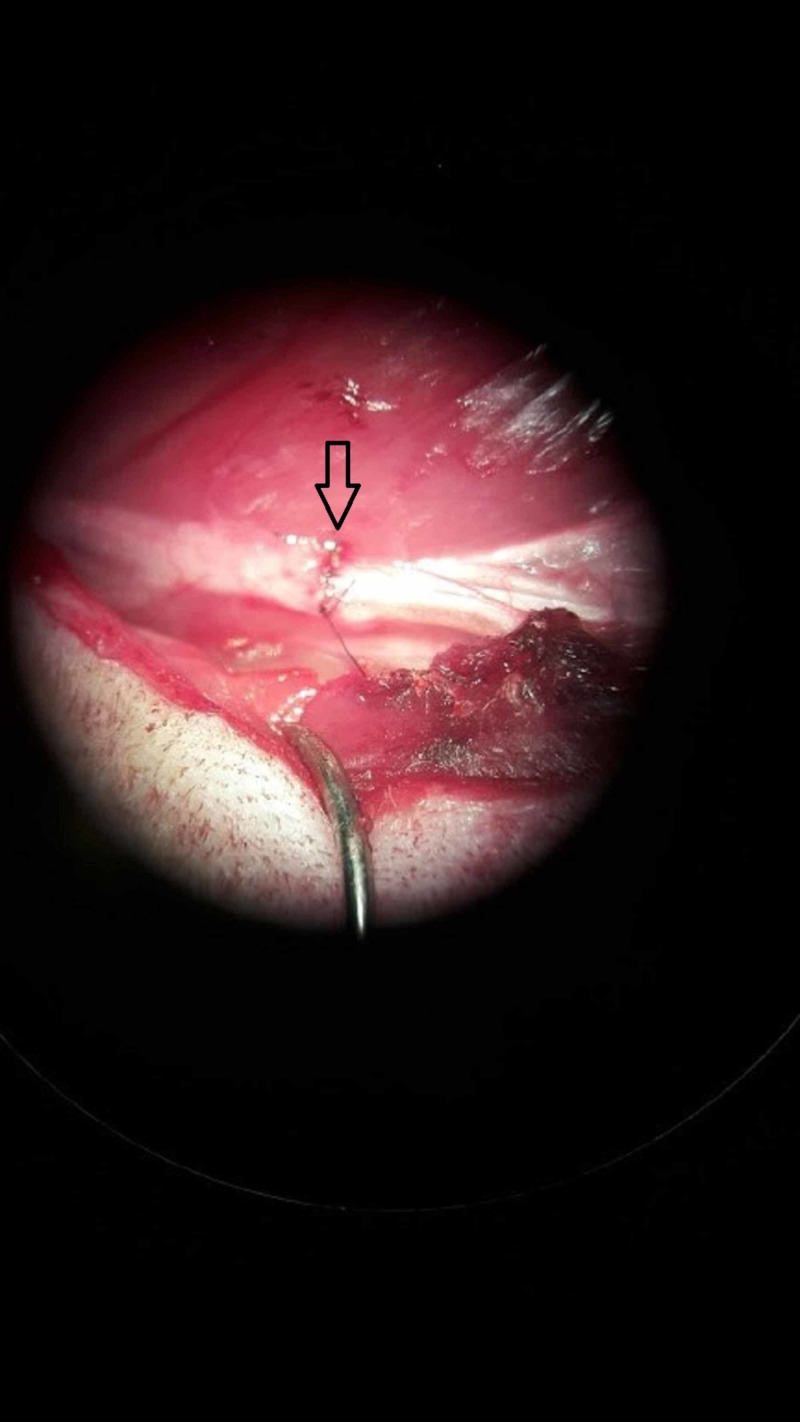
Sciatic nerve of the rat after nerve repair (microscopy x16). The arrow shows the suture repair area.

Surgery was performed to create the sciatic nerve cut model. A ketamine-medetomidine mixture (0.5 mg/kg of medetomidine and 50 mg/kg of ketamine intramuscularly) was given intraperitoneally for general anesthesia. After the rats were placed on the operation table in the Thompson position, the surgical area was prepped and draped. The sciatic nerve was exposed with an incision that started 1 cm distal to the sciatic notch and 1 cm proximal to the trifurcation of the nerve in the posterior part of the knee joint. The sciatic nerve was identified and prepared 3 to 3.5 cm proximal to its trifurcation division. The nerve was then transversely cut with the use of a micro scalpel 1.5 cm proximal to the trifurcation (Figures 1, 2). Finally, the transected nerve was repaired by the main investigator with epineural sutures (10-0 Ethilon®, Ethicon Inc., Somerville, NJ, USA) (Figure 3) under microscopy (x16). In the PRP group, 1.5 mL of blood drawn from the tail vein was collected in a citrated tube. After 7 minutes of differential centrifugation at 700 RCF, the upper portion of the volume (0.7 mL of PRP) was injected to an absorbable gelatin sponge (0.5 x 0.5 x 0.1 cm, Spongostan®, Ethicon Inc.) and applied onto the repair area. In the MSCs group, MSCs were directly collected from the trochanteric area of the femur with the use of a trocar drill. The b-MSCs were then injected onto the absorbable gelatin sponge and applied onto the nerve repair area. In the control group, normal saline 0.09% was injected onto the absorbable gelatin sponge and applied onto the repair area. The wound was closed with No 4-0 sub-cutaneous absorbable sutures (Vicryl®, Ethicon Inc.) and non-absorbable No 5-0 skin sutures (Nylon®, Ethicon Inc.). In all rats, a single dose of antibiotic, enrofloxacin (10 mg/kg of Baytril® 5%, Bayer AG, Leverkusen, Germany), was administered. Following recovery, the rats were returned to their cages and allowed to perform normal activities. All operated animals were again divided in two sub-groups: (1) the with-NSAIDs group, where meloxicam (0.05 mg/kg/24 hours) and paracetamol (1 mg/kg/12 hours) was administered for one week, and (b) the without-NSAIDs group, where only paracetamol was administered for one week. At 12 weeks post-operatively, nerve conduction studies were applied to both legs of all the animals. The animals were then sacrificed under anesthesia with bleeding of the posterior vena cava concave vein, and specimens of the repaired sciatic nerves were prepared for histological evaluation.

Nerve conduction study

All electrodiagnostic evaluations were performed blindly by the same author (P. K.) using the same EMG device. An EMG apparatus (Keypoint® Classic, Medtronics, Copenhagen, Denmark) was used for the measurements. The low- and high-pass filter settings were 20 Hz and 10 kHz, respectively. The stimulus had duration of 0.3 ms, a frequency of 0.5 Hz, and a sweep velocity of 5 ms/div. Supramaximal stimulation was used. Ring surface electrodes were used for the recordings while the rats were under general anesthesia (0.5 mg/kg of medetomidine and 50 mg/kg of ketamine intramuscularly). Subcutaneous platinum needle electrodes (Grass, Astro-Med Inc., West Warwick, RI, USA) were used for stimulation. The sciatic nerve was stimulated supramaximally at the sciatic notch point. Compound muscle action potentials (CMAP) were recorded from the gastrocnemius muscle of both legs. The latency from the stimulus to the first deflection from baseline was measured in milliseconds (ms), the CMAP amplitude from the first negative to the next positive peak in millivolts (mV), the CMAP duration from the initial to the terminal deflection back to baseline in ms, and the CMAP area in ms*mV.

Histological study

All histology preparation was supervised by the same author (K. D.), and the evaluation was blindly performed by two authors (K. D. and N. K.). The sciatic nerve was fixed in a 10% formalin buffer solution. Paraffin-embedded tissue was then sectioned into 0.3- to 0.5-mm-thick serial sections on positive charged slides. The sections were stained with hematoxylin-eosin stain and immunohistochemically evaluated with S-100 antibody (polyclonal rabbit) as a marker for myelin sheath (Dako, Glostrup, Denmark). Regarding the S100 antibody, tissues were pre-treated with heat-induced epitope retrieval (HIER) method (PT, Dako) using the Dako Envision Flex Retrieval Solution at high pH and 96-98⁰C for 20 minutes. The tissue was incubated at room temperature for 30 minutes according to the IHC immunohistochemistry protocol of Dako Envision Flex Kit, which uses diaminobenzidine as chromogen. Samples were photographed under a light microscope (Axioskop 2 Plus, Zeiss, Oberkochen, Germany). Neuronal axons were intensely immunostained with S-100 antigen, and their absolute number was measured [8]. Normal sciatic nerves from the contralateral non-operated left leg were used as control. The absolute number of axons was measured by examining six randomly selected fields (one center, five periphery) with the use of a digital counter with a x40 magnification from three sites: proximal to nerve repair, distal to nerve repair, and far distal (0.3 cm) to nerve repair. The procedure was performed both manually under light microscopy and by the ImagePro Plus® v6.0 software interface (Media Cybernetics Inc., Rockville, MD, USA) and applied in the regions of interest for neuronal axons counting and cell morphometry. All evaluations were expressed as a number of positive immunostaining per 1 μm^2^.

Statistical analysis

Data were analyzed by a biostatistician (A. G.) and expressed as mean ± standard deviation (SD) or mean ± standard error (SE) (for two-way analysis-of-variance [ANOVA] results) for continuous variables and as percentages for categorical data. The Kolmogorov-Smirnov test was used for normality analysis of the parameters. Two-way ANOVA model was used to examine the interaction between the “treatment” factor and “anti-inflammatory” factor. Since there was no statistically significant interaction, we compared the “treatment” factor regardless of the “anti-inflammatory” factor and the “anti-inflammatory” factor regardless of “treatment” factor.

The comparison of variables factor between “treatment” groups was performed for each sub-group of “anti-inflammatory” separately using the one-way ANOVA model. Pairwise comparisons were performed using the Bonferroni test. The comparison of variables between the sub-groups of “anti-inflammatory” factor for each “treatment” group was performed separately using the independent samples t-test.

All tests were two-sided, and statistical significance was set at p<0.05. All analyses were carried out using the statistical package SPSS Version 21 (IBM Corp., Armonk, NY, USA).

## Results

**Figure 4:**
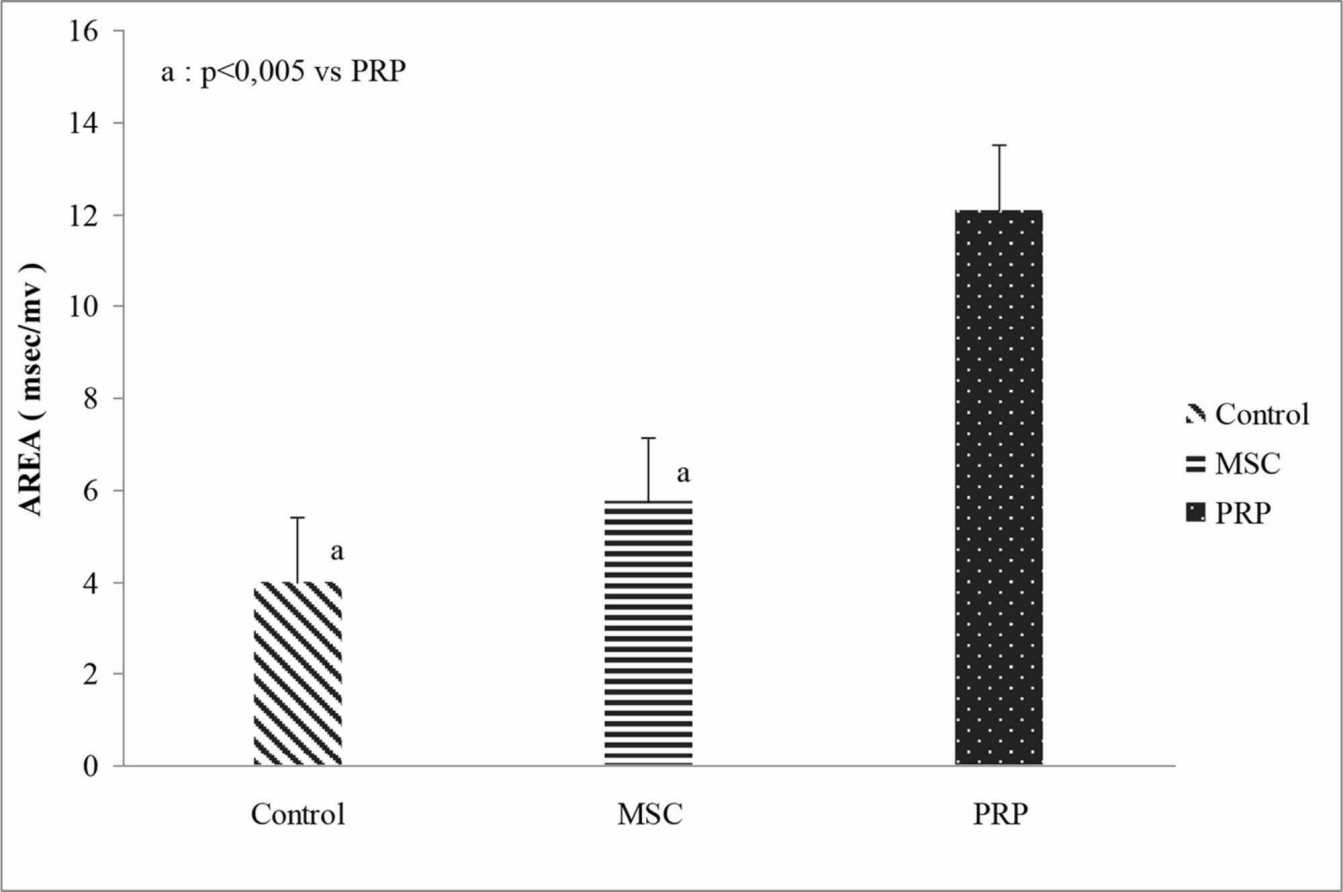
CMAP area of the operated leg between the “treatment” groups regardless of the “anti-inflammatory” sub-groups. PRP, platelet-rich plasma; MSCs, mesenchymal stem cells; CMAP, compound muscle action potentials

Concerning the CMAP area, there were statistically significant differences between the “treatment” groups (p<0.005). The PRP group showed a significantly higher CMAP area when compared with the control group (p=0.001). The MSCs group also demonstrated statistically significant better result when compared with the control group (p=0.006). Furthermore, no difference was found between the “anti-inflammatory” sub-groups regardless of the “treatment” (p=0.939) (Figure 4, Table 2). Two-way ANOVA showed no significant interaction between "treatment" and “anti-inflammatory” factors (p=0.704).

**Table 2:** CMAP area (mV/msec) of the operated leg. ^a^p<0.05 vs. the PRP group. ^b^p<0.005 vs. the PRP group. NSAIDs, non-steroidal anti-inflammatory drugs; SD, standard deviation; SE, standard error; MSCs, mesenchymal stem cells; PRP, platelet-rich plasma; CMAP, compound muscle action potentials

	Non-NSAIDs sub-group	NSAIDs sub-group	Comparison between sub-groups	Comparison between “treatment” groups regardless of “anti-inflammatory” sub-groups
	Mean±SD	Mean±SD		Mean±SE
Control	4.62±3.28^a^	3.37±2.68^b^	p=0.467	3.99±1.38^b^
MSCs	4.88±3.54^a^	6.60±5.01^a^	p=0.463	5.74±1.34^b^
PRP	12.52±8.79	11.68±4.94	p=0.823	12.10±1.34
Comparison between “treatment” groups	p=0.036	p=0.006		p<0.0005
	Mean±SE	Mean±SE		Interaction between “treatment groups and “anti-inflammatory” sub-groups, p=0.704
Comparison between “anti-inflammatory” sub-groups regardless of “treatment” groups	7.34±1.12	7.21±1.10	p=0.939	

**Figure 5:**
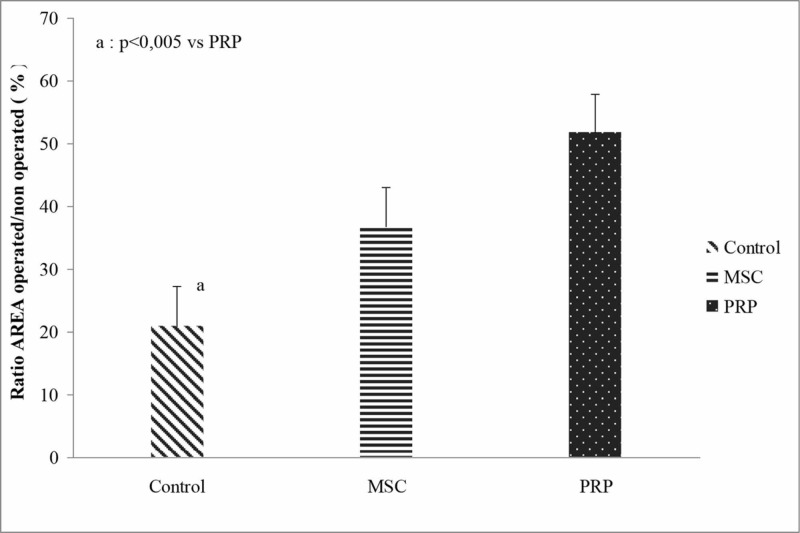
CMAP area ratio (operated leg / non-operated leg *100) between the “treatment” groups regardless of the “anti-inflammatory” sub-groups. PRP, platelet-rich plasma; MSCs, mesenchymal stem cells; CMAP, compound muscle action potentials

With respect to the CMAP ratio area% (operated/non-operated*100), there was a statistically significant difference between “treatment” groups (p=0.005). PRP group showed statistically significant better results when compared with the control group (p=0.003). Furthermore, no difference was found between the “anti-inflammatory” sub-groups regardless of “treatment” (p=0.404) (Figure 5, Table 3). Two-way ANOVA showed no significant interaction between the “treatment” and “anti-inflammatory” factors (p=0.477).

**Table 3:** Ratio of CMAP area between the operated leg and the non-operated leg. ^a^p<0.05 vs. the PRP group. ^b^p<0.005 vs. the PRP group. NSAIDs, non-steroidal anti-inflammatory drugs; SD, standard deviation; SE, standard error; MSCs, mesenchymal stem cells; PRP, platelet-rich plasma; CMAP, compound muscle action potentials

	Non-NSAIDs sub-groups	NSAIDs sub-groups	Comparison between sub-groups	Comparison between “treatment” groups regardless of “anti-inflammatory” sub-groups
	Mean±SD	Mean±SD		Mean±SE
Control	22.82±19.70	19.07±17.66^a^	p=0.724	20.94±6.26^b^
MSCs	28.18±11.16	45.61±34.27	p=0.277	36.89±6.07
PRP	49.65±24.42	53.91±24.42	p=0.752	51.78±6.07
Comparison between “treatment” groups	p=0.161	p=0.047		p=0.005
	Mean±SE	Mean±SE		Interaction between “treatment” groups and “anti-inflammatory” sub-groups, p=0.477
Comparison between “anti-inflammatory” sub-groups regardless of “treatment” groups	33.55±5.08	39.53±4.94	p=0.404	

As regards the CMAP duration, no significant differences were found between the “treatment” groups. However, the CMAP amplitude of the right gastrocnemius muscle was significantly higher for the PRP group when compared with the control group but not significant when compared with the non-operated (intact) left leg.

**Figure 6:**
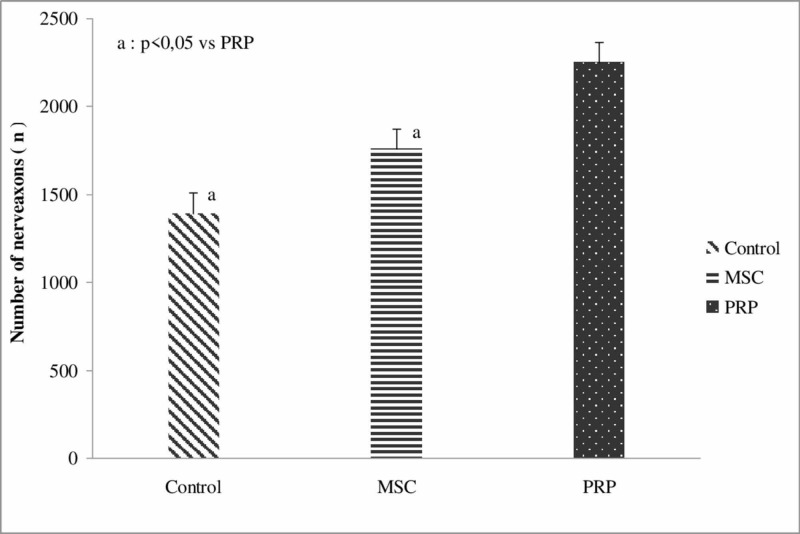
Absolute number of nerve axons distal to nerve repair between the “treatment” groups regardless of the “anti-inflammatory” sub-groups. PRP, platelet-rich plasma; MSCs, mesenchymal stem cells

The absolute number of neural axons (number/μm^2^) distal to nerve repair was significantly higher in PRP group when compared to the control group (p=0.005). However, it was not the same in the MSCs group when compared with the PRP group (Figure 6).

**Figure 7:**
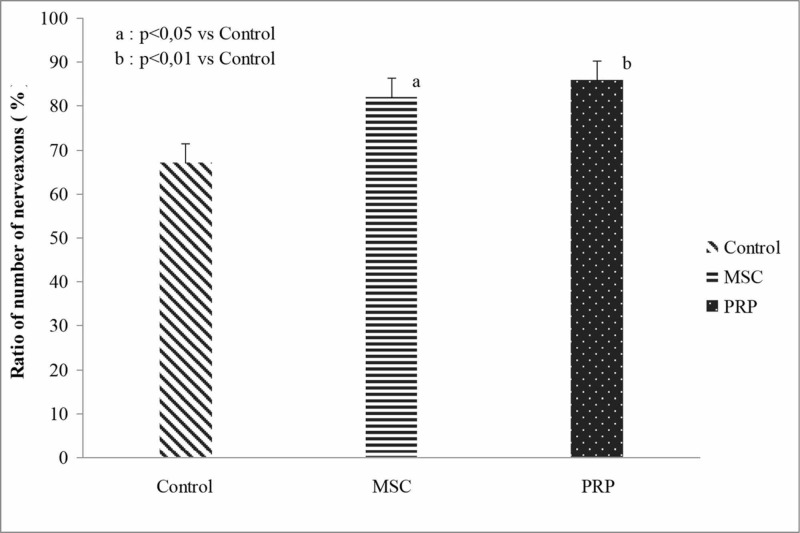
DP ratio of the average number of neuronal axons between the “treatment” groups regardless of the “anti-inflammatory” sub-groups. PRP, platelet-rich plasma; MSCs, mesenchymal stem cells; DP, distal nerve repair to proximal nerve repair

The ratio of the number of axons distal to nerve repair and the number of axons proximal to nerve repair (D/P) was significantly higher in the PRP and MSCs groups when compared with the control group (p=0.009 and p=0.047, respectively). No difference was found between the “anti-inflammatory” sub-groups regardless of the treatment (Figure 7, Table 4). The two-way ANOVA result showed no significant interaction between the “treatment” and “anti-inflammatory factors” (p=0.822).

**Table 4:** DP ratio. ^a^p=0.047 vs. the control group. ^b^p=0.009 vs. the control group.

	Non-NSAIDs sub-groups	NSAIDs sub-groups	Comparison between sub-groups	Comparison between “treatment” groups regardless of “anti-inflammatory” sub-groups
	Mean±SD	Mean±SD		Mean±SE
Control	62.76±12.42	71.14±16.50	p=0.331	66.9±4.4
MSCs	79.97±19.80	85.16±15.53	p=0.606	82.6±4.3^a^
PRP	86.28±15.95	86.98±12.96	p=0.929	86.7±4.3^b^
Comparison between “treatment” groups	p=0.066	p=0.119		p=0.007
	Mean±SE	Mean±SE		Interaction between “treatment” groups and “anti-inflammatory” sub-groups, p=0.822
Comparison between “anti-inflammatory” sub-groups regardless of “treatment” groups	76.3±3.58	81.1±3.48	p=0.348	

**Figure 8:**
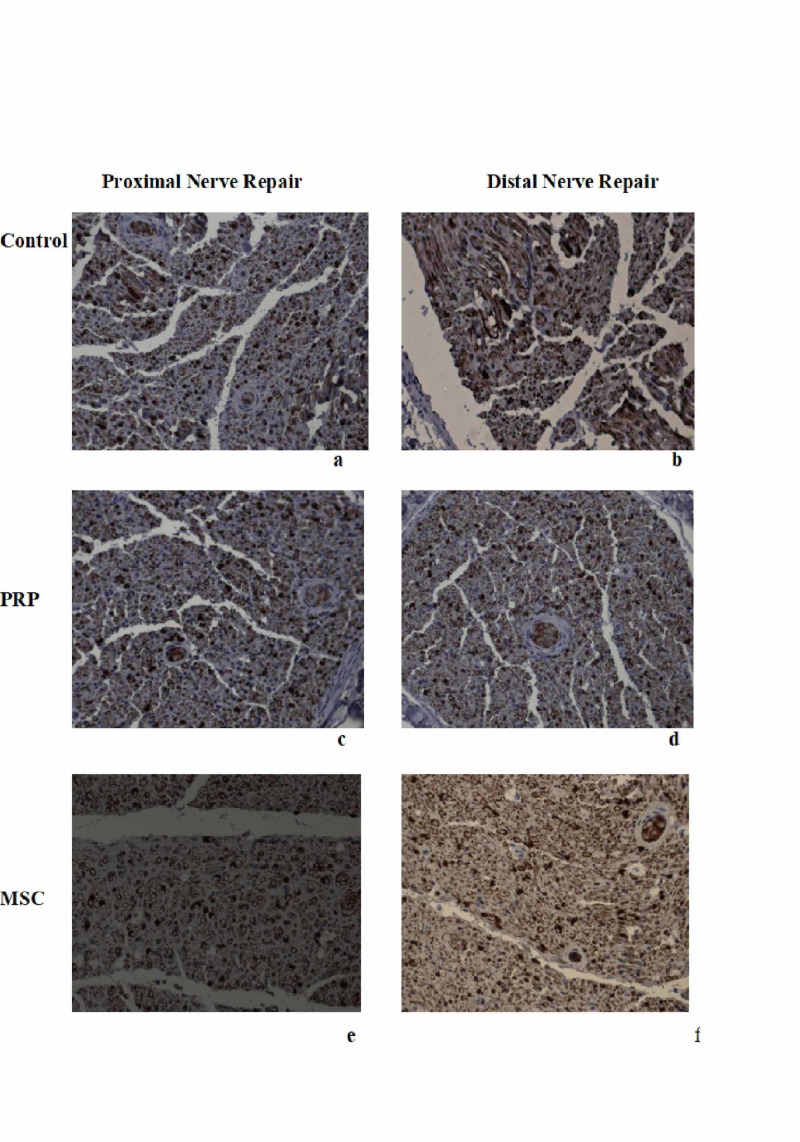
Histology under x40 magnification proximal and distal to nerve repair of the three “treatment” groups. (a) Control group proximal to nerve repair. (b) Control group distal to nerve repair. (c) PRP group proximal to nerve repair. (d) PRP group distal to nerve repair. (e) MSCs group proximal to nerve repair. (f) MSCs group distal to nerve repair. PRP, platelet-rich plasma; MSCs, mesenchymal stem cells

With regard to the diameter of the newly formed neuroaxons, there was no significant difference between the “treatment” groups or the “anti-inflammatory” sub-groups (Figure 8).

## Discussion

Microsurgical suturing techniques for fine nerve repair have witnessed impressive development in recent years. Furthermore, bio-artificial structures called “conduits” serve as an alternative to autologous nerve grafts, with the aim to bridge the defects in nerve discontinuity [9]. However, these methods failed to constantly reproduce satisfactory results for nerve repair [2]. Despite extensive studies in the field of nerve regeneration, the molecular mechanisms underlying the post-traumatic processes in nerve fibers remain poorly understood and require further investigation [10]. Adjuvant biologic treatment with local application of PRP or MSCs on the nerve repair site remains a wide-open field for research. The results of the present experimental animal study showed that enrichment of PRP or MSCs on nerve repair resulted in better functional recovery of the sciatic nerve with better structural indexes on regenerated nerve mainly on the distal part of the repair, indicating that biological adjuvant therapies may play a role in the peripheral nerve regeneration.

Ding et al. in their study of 24 rats applied local PRP after creating crush injury on the cavernous nerve [11]. The authors concluded that the functional healing and histological parameters in the PRP group were significantly better than that in the placebo group. A similar study in which a crush injury was created on the sciatic rat nerve also showed positive effects of PRP on nerve regeneration [12]. Sariguney et al. concluded that PRP is effective when applied following the ideal surgical nerve repair in a nerve transection model and was ineffective in cases of insufficient surgical repair [13]. In contrast to the above positive effects of PRP, recent studies reported unsatisfactory results. Welch et al. reported no significant effect of PRPs on a transection and direct repair on a rat model [14]. Piskin et al. concluded that PRPs did not improve nerve regeneration after microsurgical reconstruction of a nerve gap using collagen tubes [4]. As a cellular carrier, two studies in an acute nerve injury model in guinea pigs and rabbits applied PRPs and seeded the acellular carrier with MSCs, and reported beneficial effects on axonal counts, myelination, and electrophysiological parameters [15,16]. PRPs have also been used as a filler of acellular nerve allografts [17]. They showed a dose-dependent effect on the proliferation, migration, and neurotrophic function in rat ΜSCs cultured with PRP. They also showed significant improvement in the diameter, thickness, and number of myelinating axons, as well as an enhancement of electrophysiological parameters in sciatic nerve injury repaired with autografts and PRP in a rat model [18].

Using platelet-rich fibrin (PRF) as a filler of silicon nerve guidance or nerve grafts in a rat model, animals treated with PRPs showed improved functional recovery and a superior sciatic functional index compared with non-treated animals [19,20]. However, the researchers did not find morphometric or structural improvements. The application of PRPs as a fibrin membrane to wrap the neurorrhaphy in an acute injury model of sciatic nerve neurotmesis showed diverse positive effects [21]. The authors observed a stronger EMG signal, a significantly larger axonal density, and a lower scar tissue in animals treated with PRP fibrin membranes, and remains of PRP membranes were still present after six weeks post-surgery. In this sense, two studies reported the positive effects of using PRP as adjuvant treatment in nerve suturing. Farrag et al. reported that PRPs may enhance the myelin thickness and increase the axon counts when the injured nerve is sutured and assisted with PRP, whereas Sariguney et al. found no positive effects on axonal size in sutured nerves enriched with PRP. However, they showed a better functional outcome associated with improvement in the myelin thickness and the onset latency [13,22].

Regenerative cell therapy pathways that influence a successful outcome after a nerve repair are still poorly understood. These biological mechanisms could be classified into the following groups: (1) differentiation toward the Schwann cell lineage, (2) contribution to myelination of regenerating axons, (3) production of trophic factors and extracellular matrix proteins that provide a milieu for axonal outgrowth, (4) stimulation of proliferation and differentiation of endogenous cells, (5) stimulation of angiogenesis, and (6) immunosuppression. The analysis of the molecular mechanisms underlying the interaction between MSCs and immune cells demonstrated that MSCs suppress T and B lymphocytes and inhibit dendrite cell maturation [23]. MSCs enhances axon regeneration not only when delivered to the injured nerve or conduit bridging the nerve gap but also when administered intravenously [6,7]. Furthermore, Tomita et al. performed in vitro and in vivo studies of glial differentiation of MSCs derived from human adipose tissue. It was established that following exposure to glial growth factors, MSCs transdifferentiate into a Schwann cell phenotype. Lineage-committed MSCs demonstrated a seven-fold higher survival rate after implantation than multipotent MSCs in a rat tibial nerve injury study [24].

Non-steroid anti-inflammatory drugs (NSAIDs) administration seems to positively affect nerve regeneration. Madura et al. showed that ibuprofen significantly enhanced regeneration after tibial nerve axonotomy and repair in a rat model [25]. Sharp et al. showed that their results only partially replicate the findings that the treatment with ibuprofen improves motor function after spinal cord injury but failed to replicate findings regarding enhanced axon growth [26]. Moreover, an immunohistochemical study showed a more positive location of reactions to S-100 in the group loaded with diclofenac in an artery graft than the group in which an artery graft was buffered with saline alone. Therefore, the diclofenac improved functional recovery and morphometric indices of the sciatic nerve [8]. However, in our study, despite current literature, systematic NSAIDs administration did not show any additional positive effect on nerve regeneration.

Limitations

There were a few limitations in this study. We performed a single centrifugation process and produced leukocyte-rich PRPs rather than pure PRP (p-PRP). The b-MSCs were immediately applied to the nerve repair site instead of culturing them up to a high concentration of pure MSCs. However, as mentioned earlier, in clinical practice when a nerve injury is diagnosed, a nerve repair is an urgent situation and there is no time for p-PRP preparation or MSCs cultivation. Advantages of our study were (1) the use of contralateral leg to adjust the EMG values between injured and non-injured leg for each animal and (2) the use of a statistically significant number of subjects following power analysis of the protocol.

## Conclusions

In this study, the intra-operative administration of PRP or MSCs following a sciatic nerve repair improved histology architecture and enhanced functional outcome compared to the control groups. The results of this study are encouraging for clinical trials in humans supporting the findings of other recent studies. Local intra-operative single-dose administration of either PRP or MSCs on the repair site of a damaged nerve seems to enhance tissue regeneration in terms of histological findings and therefore may ameliorate the final functional outcome in terms of EMG findings. In clinical practice, the healing process of an acutely repaired peripheral nerve may benefit from adjuvant biological therapy. Thus, the intra-operative use of autologous biological substances such as PRPs obtained from peripheral veins or MSCs harvested from cancellous bone marrow is a safe, low-cost, fast, single-stage technique that may enhance the nerve reparative procedure and the final functional outcome.
